# Etude structurale et vibrationnelle d’un nouveau composé complexe de cobalt: [Co(imidazole)_4_Cl]Cl

**DOI:** 10.1107/S2056989015015807

**Published:** 2015-09-17

**Authors:** Amira Derbel, Tahar Mhiri, Mohsen Graia

**Affiliations:** a Laboratoire de l’état solide, Faculté des Sciences, Université de Sfax, BP W 3038 Sfax, Tunisie; b Laboratoire de Matériaux et Cristallochimie, Faculté des Sciences de Tunis, Université de Tunis El Manar, 2092 El Manar Tunis, Tunisie

**Keywords:** crystal structure, cobalt complex, imidazole, hydrogen bonding, framework

## Abstract

In the title complex, chlorido­tetra­kis­(1*H*-imidazole-κ*N* ^3^)cobalt(II) chloride, [CoCl(C_3_H_4_N_2_)_4_]Cl, the Co^II^ cation has a distorted square-pyramidal coordination environment. It is coordinated by four N atoms of four imidazole (Im) groups in the basal plane, and by a Cl atom in the apical position. It is isostructural with [Cu(Im)_4_Cl]Cl [Morzyk-Ociepa *et al.* (2012 ▸). *J. Mol. Struct.* **1028**, 49–56] and [Cu(Im)_4_Br]Br [Hossaini Sadr *et al.* (2004 ▸). *Acta Cryst*. E**60**, m1324–m1326]. In the crystal, the [CoCl(C_3_H_4_N_2_)_4_]^+^ cations and Cl^−^ anions are linked *via* N—H⋯Cl hydrogen bonds, forming layers parallel to (010). These layers are linked *via* C—H⋯Cl hydrogen bonds and C—H⋯π and π–π [inter-centroid distance = 3.794 (2) Å] inter­actions, forming a three-dimensional framework. The IR spectrum shows vibrational bands typical for imidazol groups. The monoclinic unit cell of the title compound emulates an ortho­rhom­bic cell as its β angle is close to 90°. The crystal is twinned, with the refined ratio of twin components being 0.569 (1):0.431 (1).

## Abstract 2

In the title complex, the Co^II^ cation has a distorted square-pyramidal coordination environment, being coordinated by four N atoms of four imidazole groups in the basal plane and by a Cl atom in the apical position. In the crystal, the [CoCl(C_3_H_4_N_2_)_4_]^+^ cations and chloride Cl^−^ anions are linked *via* N—H⋯Cl hydrogen bonds, forming layers parallel to (010).

## Contexte chimique   

L’imidazole et ses dérivés sont considérés parmi les hétérocycles les plus intéressants qui peuvent participer à la formation d’ions complexes. Ils se retrouvent couramment dans plusieurs molécules naturelles telles l’acide aminé histidine, la caféine, les purines ou la vitamine B12. En biologie, il s’agit d’un pharmacophore dont la toxicité est plutôt faible (OECD, 2003 ▸). Ainsi, le motif imidazole figure dans la formulation de nombreux médicaments, notamment des anti-inflammatoires, anti­fongiques, anti­hypertenseurs et même anti­cancéreux (Shargel *et al.*, 2006 ▸; Castaño *et al.*, 2008 ▸; Bogle *et al.*, 1994 ▸).
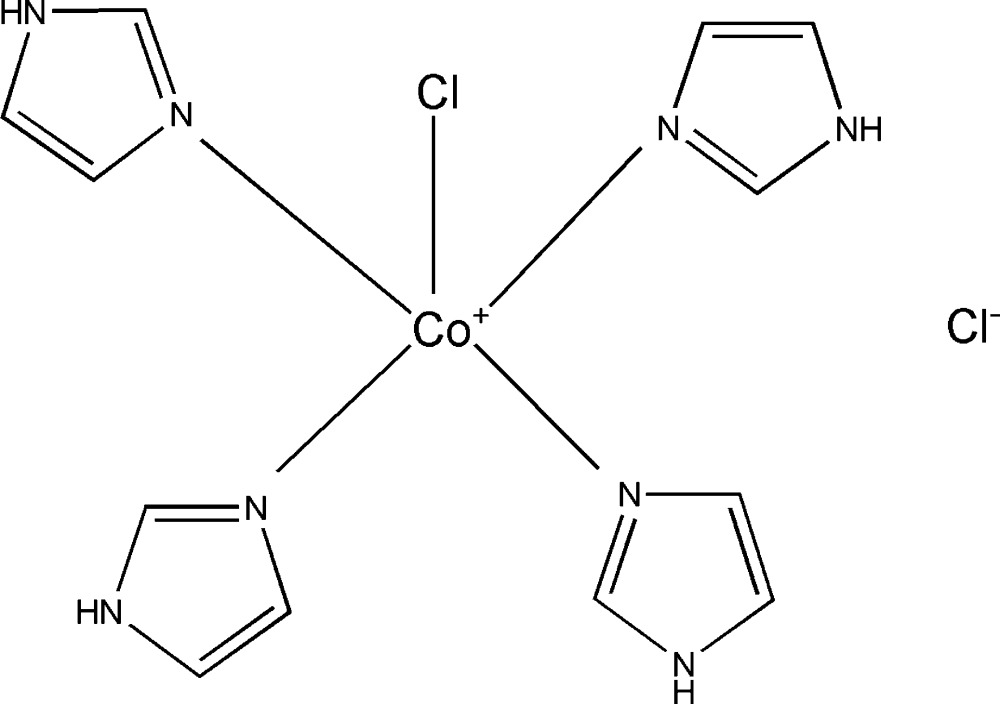


## Commentaire structurelle   

**Figure 1:**
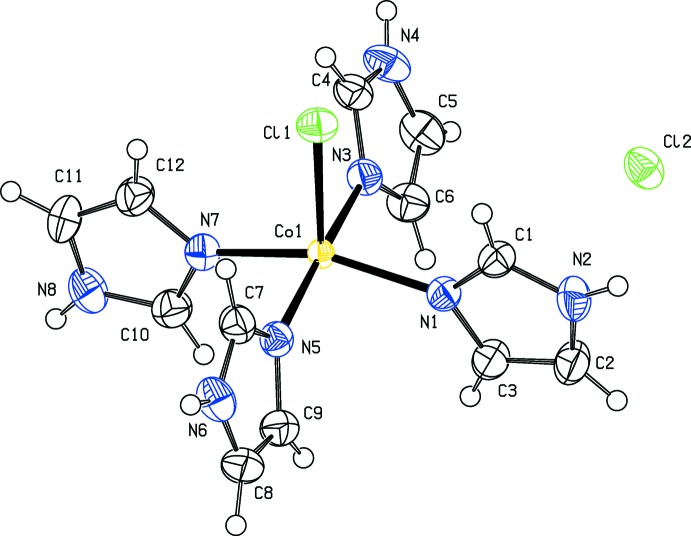
Unité asymétrique du composé [Co(imidazole)_4_Cl]Cl. Les éllipsoïdes ont été définis avec 50% de probabilité.

L’unité asymétrique du composé étudié [Co(C_3_H_4_N_2_)Cl]Cl est formée d’un cation complexe [Co(C_3_H_4_N_2_)Cl]^+^ et d’un anion chlorure libre Cl^−^ (Fig. 1 ▸). L’ion complexe [Co(C_3_H_4_N_2_)Cl]^+^ est formé par un cation Co^2+^ coordiné à quarte molécules imidazoles par des liaisons Co—N et un seul anion chlorure (Co—Cl1). Ceci conduit à un environnement pyramide à base carrée (penta­èdre) autour du cation Co^2+^ (Fig. 1 ▸ et tableau 1 ▸). Le détail de la structure montre que le polyèdre de coordination du cobalt CoN_4_Cl est une pyramide distordue. En effet, la distance Co—Cl est de 2,615 (6) Å et les distances Co—N varient de 1.999 (2) à 2.011 (2) Å. En plus, les angles Cl—Co—N ont des valeurs variables entre 104,28 (7) et 92,40 (8)°, les angles *trans* N—Co—N sont de 157,60 (9) et 174,57 (9) ° et les angles *cis* N—Co—N varie de 88,92 (11) à 90,26 (11) °. Les penta­èdres CoN_4_Cl sont isolés les uns des autres avec une distance minimale Co—Co = 7,3997 (7) Å. Cette distance est supérieure à celle qui permet de prévoir un couplage magnétique (Decaroli *et al.*, 2015 ▸). Les groupements imidazoles sont plans avec des déviations standards par rapport aux plans moyens inferieures à 0,3%. Les distances C—N varient de 1,314 (4) à 1,379 (4) Å et les distances C—C varient de 1,334 (4) à 1,357 (4) Å. Ces caractéristiques géométriques sont comparables à celles observées dans les composés analogues. Enfin, on note que la maille élémentaire du composé étudié présente un volume légèrement supérieur à celui du composé isoformulaire [Cu (C_3_H_4_N_2_)_4_Cl]Cl, cet écart est de 1,3% et peut être expliqué par le rayon du cation Co^2+^ qui est supérieur à celui du cation Cu^2+^ [*R*(Co^2+^) = 0,67 Å et *R*(Cu^2+^) = 0,65 Å; Shannon, 1976 ▸].

## Caractéristiques supra­moléculaires   

**Figure 2:**
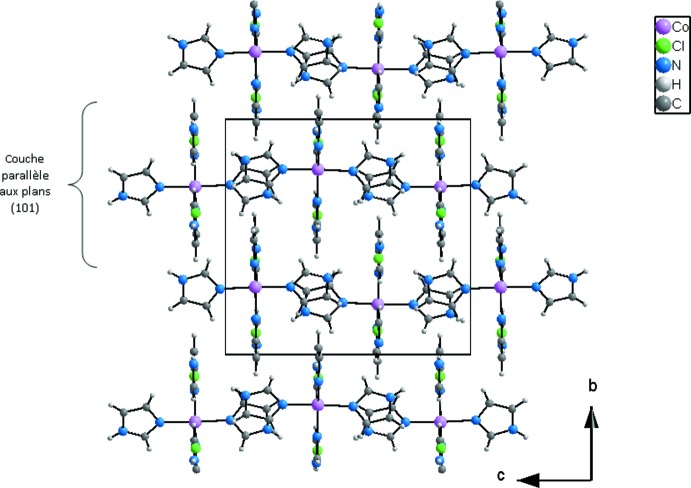
Projection de la structure du composé [Co(imidazole)_4_Cl]Cl dans le plan *bc* montrant l’arrangement en couches.

**Figure 3:**
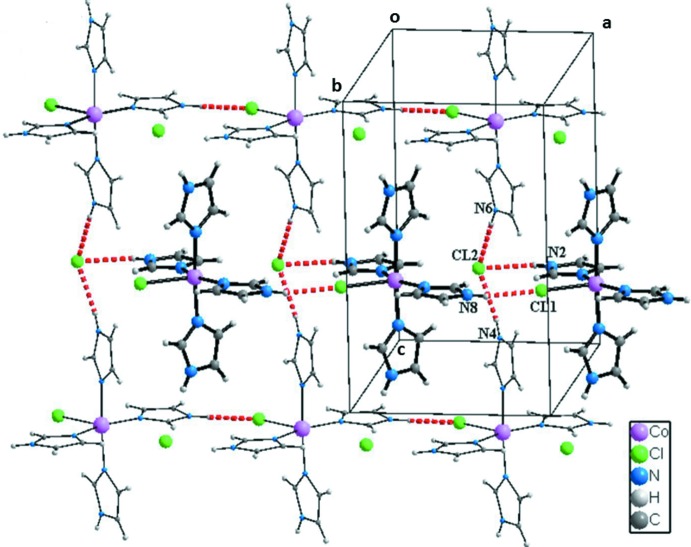
Cohésion dans une couche du composé [Co(imidazole)_4_Cl]Cl par des liaisons hydrogène N—H⋯Cl.

**Figure 4:**
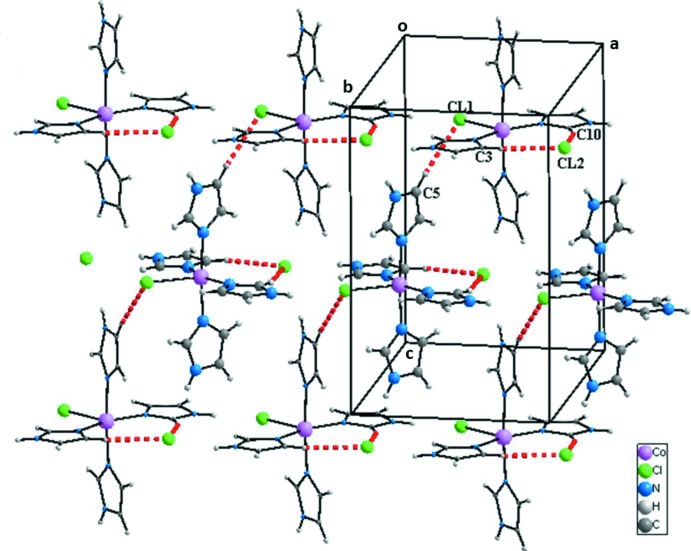
Cohésion dans une couche du composé [Co(imidazole)_4_Cl]Cl par des liaisons hydrogène C—H⋯Cl.

**Figure 5:**
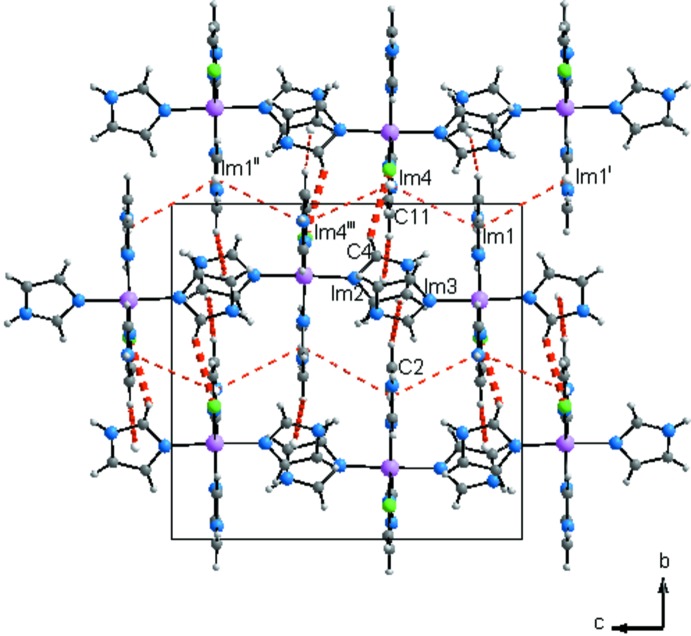
Liaisons entre couches dans la structure du composé [Co(imidazole)_4_Cl]Cl. [Codes de symétrie: (i) −*x* + 3, −*y* + 2, −*z*; (ii) −*x* + 1, −*y* + 2, −*z* + 1; (iii) −*x* + 1, −*y* + 2, −*z* + 1.]

La structure du composé peut être décrite comme une succession de couches parallèles au plan (010) et imbriquées les unes dans les autres (Fig. 2 ▸). Dans chacune des couches les ligands Cl1 permettent la connexion des groupements complexes en rubans infinis par des liaisons hydrogène N8—H8*N*⋯Cl1 (Fig. 3 ▸ et tableau 2 ▸). Les anions Cl2^−^ se situent dans ces rubans et permettent la cohésion dans la couche. En effet chacun de ces anions établi trois liaisons hydrogène N2—H2*N*⋯Cl2, N4—H4*N*⋯Cl2 et N6—H6*N*⋯Cl2 engageant un cation complexe du même ruban et deux autres situés dans des rubans adjacents (Fig. 3 ▸ et table 2 ▸). En plus des liaisons hydrogène relativement fortes N—H⋯Cl, d’autres liaisons de type C—H⋯Cl, de plus faibles énergies contribuent à la cohésion dans les couches. En effet, l’anion Cl1 établi une liaison C4—H4⋯Cl1 engageant deux rubans adjacents alors que l’anion Cl2 établi deux liaisons C3—H3⋯Cl2 et C10—H10⋯Cl2 appartenant à un même groupement complexe adjacent du même ruban (Fig. 4 ▸ et tableau 2 ▸). La Fig. 5 ▸ (et tableau 2 ▸), montre que la connexion entre couches fait inter­venir des liaisons hydrogène de faibles énergies C4—H4⋯Cl1 et C2—H2⋯Im3 en plus des inter­actions π–π faisant inter­venir les groupements imidazoles Im1⋯Im4 [3,914 (2) Å], Im4⋯Im4^iii^ [3,794 (2) Å] et Im4^iii^⋯Im1^ii^ [3,914 (2) Å], avec Im = centroïde du cycle imidazole; Im1: N1/N2/C1–C3; Im4: N7/N8/C10–C12; pour les opérations de symétrie voir Fig. 5 ▸).

## Enquête de base de données   

L’étude structurale déjà effectuée (Morzyk-Ociepa *et al.*, 2012 ▸) a mis en évidence le polymorphisme des complexes de cuivre [Cu(Im)_4_Cl]Cl en identifiant deux formes structurales qu’ils ont noté (1) et (2). Cette étude est la seule, à nôtre connaissance, qui a évoquée la présence de la forme (2) des composés [*M*(Im)_4_ *X*]*X* (avec *M* = métal de transition et *X* = Cl, Br). Cependant, la forme (1) à fait l’objet au moins de quatre études structurales relatives aux composés [Cu(Im)_4_Cl]Cl (Morzyk-Ociepa *et al.*, 2012 ▸; Li *et al.*, 2007 ▸; Wu *et al.*, 2013 ▸) et [Cu(Im)_4_Br]Br (Hossaini Sadr *et al.*, 2004 ▸). En plus, un examen bibliographique montre que six composés complexes analogues et de formulation générale [*M*(*R*-Im)_4_ *X*]*X* (avec *M* = métal de transition, *R* = groupement alkyle et *X* = Cl, Br) sont identifiés (Godlewska *et al.*, 2011*a* ▸,*b* ▸, 2012 ▸, 2013 ▸; Atria *et al.*, 2003 ▸; Liu *et al.*, 2007 ▸). Dans la totalité des composés étudiés déjà cités, le métal central est le cuivre. Le présent travail est consacré à l’étude du complexe du cobalt [Co(Im)_4_Cl]Cl, isotype à la forme (1) du complexe de cuivre analogue.

## Etude vibrationnelle   

**Figure 6:**
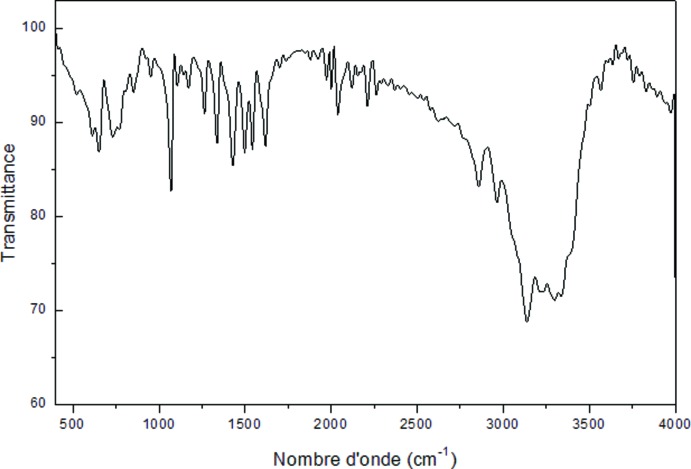
Spectre d’absorption infra rouge à la température ambiante du composé [Co(imidazole)_4_Cl]Cl.

Le spectre IR de ce composé a été enregistré dans le domaine de fréquences qui s’étend de 400 à 4000 cm^−1^ (Fig. 6 ▸). L’attribution des modes inter­nes et externes est basée sur la comparaison avec les composés de la littérature et des composés similaires (Moryk-Ociepa *et al.*, 2012 ▸; Wu *et al.*, 2013 ▸). Les bandes très larges qui apparaissent entre 3310 cm^−1^ et 3120 cm^−1^ correspondent à la vibration de valence de groupement N—H, une série de bandes observée entre 2970 et 1975 cm^−1^ est due à la vibration d’élongation des groupements C—H. Les bandes de déformation de N—H se manifeste entre 1625 cm^−1^ et 1437 cm^−1^ alors que les vibrations de valence de C—C et C—N sont observées entre 1625 cm^−1^ et 1078 cm^−1^. Les pics détectés vers 950 cm^−1^ et 847 cm^−1^ sont attribués à la déformation des cycles imidazole, alors que ceux observés vers 783 et 737 cm^−1^ sont assignés aux vibrations π CH.

## Synthèse et cristallisation   

**Figure 7:**
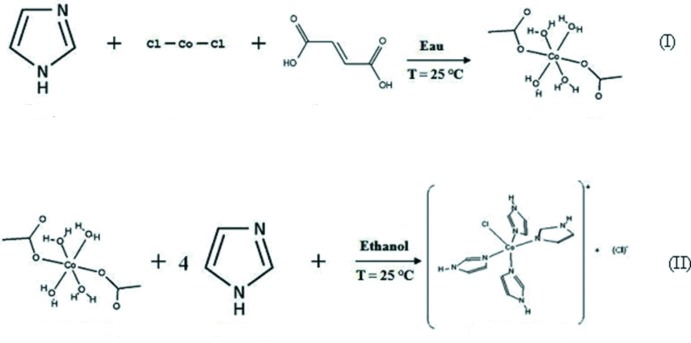
Schéma réactionnel des deux étapes de la préparation du composé [Co(imidazole)_4_Cl]Cl.

Le composé complexe de cobalt de formule [Co(imidazole)_4_Cl]Cl n’a pu être obtenu en une seule étape à partir des précurseurs chlorure de cobalt, et imidazole. L’obtention de ces cristaux à nécessité deux étapes (Fig. 7 ▸). La première étape (I) consiste à préparer des cristaux de [Co(H_2_O)_4_(C_4_H_2_O_4_)] à partir d’un mélange équimolaire de chlorure de cobalt, d’acide fumarique et d’imidazole en solution aqueuse. La solution obtenue, abandonnée quelques jours à la température ambiante, donne après évaporation lente des cristaux rose transparents en forme de prisme. L’étude structurale sur monocristal à montré que cette phase est déjà étudiée (Zheng & Xie, 2004 ▸). Dans une deuxième étape (II), une solution est préparée par dissolution des cristaux de ce composé intermédiaire [Co(H_2_O)_4_(C_4_H_2_O_4_)] et d’imidazole dans de l’éthanol absolu. Le rapport stoechiométrique de ces précurseurs est 1:4, respectivement. La solution obtenue, donne après deux semaines d’évaporation lente et à la température ambiante, des cristaux bleu transparents en forme de prisme.

## Affinement   

Les données cristallines, les conditions de la collecte des intensités et les résultats de l’affinement final sont consignées dans le tableau 3 ▸. L’isotypie de ce composé à celui du cuivre à facilité l’attribution des pics. Malgré la localisation des différents groupements de la structure plusieurs anomalies ont été observées: facteurs de reliabilités élevés (*R* = 0,212 et *Rw* = 0,516), pics résiduels intenses [(Δρ)_max_ = 6,05 e/Å^3^ et (Δρ)_min_ = −2,40 e/Å^3^] et GOF = 7,80. Ces anomalies sont interprétées comme signe de macle dans le cristal étudié. Cette macle est prévisible puisque la maille est monoclinique avec un paramètre β ≃ 90°. L’utilisation de l’instruction TWIN mise en oeuvre par le logiciel *SHELXL2013* (Sheldrick, 2015 ▸) à confirmé cette hypothèse avec un coefficient BASF = 0,431 (1). L’affinement de ce modèle structural à permis d’éliminer toutes les anomalies déjà citées [*R* = 0,027, *Rw* = 0,072, (Δρ)_max_ = 0,40 e/Å^3^, (Δρ)_min_ = −0,29 e/Å^3^ et GOF = 1,09]. La localisation des atomes d’hydrogène est effectuée sur la base de considérations géométriques et affinés en imposant des contraintes à la distance et à l’agitation thermique: C—H = 0,93 Å, N—H = 0,86 Å avec *U*_iso_(H) = 1,2*U*_eq_(C,N).

## Supplementary Material

Crystal structure: contains datablock(s) global, I. DOI: 10.1107/S2056989015015807/su5189sup1.cif

Structure factors: contains datablock(s) I. DOI: 10.1107/S2056989015015807/su5189Isup2.hkl

CCDC reference: 1420121

Additional supporting information:  crystallographic information; 3D view; checkCIF report

## Figures and Tables

**Table 1:** Paramtres gomtriques slectionns (, )

Co1N3	1,999(2)	Co1N1	2,011(2)
Co1N5	2,001(2)	Co1Cl1	2,6151(7)
Co1N7	2,003(2)		
			
N3Co1N5	174,57(9)	N7Co1N1	157,60(9)
N3Co1N7	89,27(12)	N3Co1Cl1	93,01(9)
N5Co1N7	88,92(11)	N5Co1Cl1	92,40(8)
N3Co1N1	89,46(11)	N7Co1Cl1	104,28(7)
N5Co1N1	90,26(11)	N1Co1Cl1	98,13(7)

**Table 2:** Gomtrie des liaisons hydrognes (, ) Im3 = centrode du cycle imidazole N5/N6/C7C9.

*D*H*A*	*D*H	H*A*	*D* *A*	*D*H*A*
N2H2*N*Cl2	0,86	2,53	3,180(3)	133
N4H4*N*Cl2^i^	0,86	2,40	3,247(3)	168
N6H6*N*Cl2^ii^	0,86	2,40	3,251(3)	169
N8H8*N*Cl1^iii^	0,86	2,44	3,254(3)	158
C3H3Cl2^iii^	0,93	2,78	3,639(3)	154
C4H4Cl1^iv^	0,93	2,78	3,541(4)	140
C10H10Cl2^iii^	0,93	2,77	3,657(3)	160
C2H2Im3^v^	0,93	2,82	3,526(3)	134

Codes de symtrie: (i) 
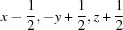
; (ii) 
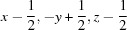
; (iii) 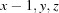; (iv) 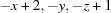; (v) 
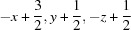
.

**Table 3:** Dtails exprimentaux

Donnes crystallines	
Formule chimique	[CoCl(C_3_H_4_N_2_)_4_]Cl
*M*_r_	402,16
Systme cristallin, groupe d’espace	Monoclinique, *P*2_1_/*n*
Temprature (K)	293
*a*, *b*, *c* ()	8,8665(3), 13,3043(6), 13,9154(5)
()	89,998(2)
*V* (^3^)	1641,50(11)
*Z*	4
Type de rayonnement	Mo *K*
(mm^1^)	1,38
Taille des cristaux (mm)	0,22 0,12 0,10

Collection de donnes	
Diffractomtre	Bruker SMART CCD area detector
Correction d’absorption	Multi-scan (*SADABS*; Bruker, 1998 ▸)
*T*_min_, *T*_max_	0,840, 0,882
Nombre de rflexions mesures, indpendantes et observes [*I* > 2(*I*)]	2915, 2915, 2705
*R*_int_	0,023
(sin /)_max_ (^1^)	0,618

Affinement	
*R*[*F* ^2^ > 2(*F* ^2^)], *wR*(*F* ^2^), *S*	0,027, 0,072, 1,09
Nombre de rflexions	2915
Nombre de paramtres	209
Traitement des atome H	H-atom paramtres contraints
_max_, _min_ (e ^3^)	0,40, 0,29

Programmes informatiques: *SMART* et *SAINT* (Bruker, 1998 ▸), *SHELXS97* (Sheldrick, 2008 ▸), *SHELXL2013* (Sheldrick, 2015 ▸), *DIAMOND* (Brandenburg Putz, 1999 ▸), *WinGX* (Farrugia, 2012 ▸) et *PLATON* (Spek, 2009 ▸).

## supplementary crystallographic information

### Crystal data

**Table:**

[CoCl(C_3_H_4_N_2_)_4_]Cl	*Z* = 4
*M**_r_* = 402.16	*F*(000) = 820
Monoclinic, *P*2_1_/*n*	*D*_x_ = 1.627 Mg m^−^^3^
*a* = 8.8665 (3) Å	Mo *K*α radiation, λ = 0.71073 Å
*b* = 13.3043 (6) Å	µ = 1.38 mm^−^^1^
*c* = 13.9154 (5) Å	*T* = 293 K
β = 89.998 (2)°	Prism, blue
*V* = 1641.50 (11) Å^3^	0.22 × 0.12 × 0.10 mm

### Data collection

**Table:**

Bruker SMART CCD area-detector diffractometer	2705 reflections with *I* > 2σ(*I*)
φ and ω scans	*R*_int_ = 0.023
Absorption correction: multi-scan (*SADABS*; Bruker, 1998)	θ_max_ = 26.1°, θ_min_ = 2.1°
*T*_min_ = 0.840, *T*_max_ = 0.882	*h* = −10→10
2915 measured reflections	*k* = −16→16
2915 independent reflections	*l* = 0→16

### Refinement

**Table:**

Refinement on *F*^2^	0 restraints
Least-squares matrix: full	Hydrogen site location: inferred from neighbouring sites
*R*[*F*^2^ > 2σ(*F*^2^)] = 0.027	H-atom parameters constrained
*wR*(*F*^2^) = 0.072	*w* = 1/[σ^2^(*F*_o_^2^) + (0.040*P*)^2^ + 0.4321*P*] where *P* = (*F*_o_^2^ + 2*F*_c_^2^)/3
*S* = 1.09	(Δ/σ)_max_ < 0.001
2915 reflections	Δρ_max_ = 0.40 e Å^−^^3^
209 parameters	Δρ_min_ = −0.29 e Å^−^^3^

### Special details

**Table:**

Geometry. All e.s.d.'s (except the e.s.d. in the dihedral angle between two l.s. planes) are estimated using the full covariance matrix. The cell e.s.d.'s are taken into account individually in the estimation of e.s.d.'s in distances, angles and torsion angles; correlations between e.s.d.'s in cell parameters are only used when they are defined by crystal symmetry. An approximate (isotropic) treatment of cell e.s.d.'s is used for estimating e.s.d.'s involving l.s. planes.
Refinement. Refined as a 2-component twin.

### Fractional atomic coordinates and isotropic or equivalent isotropic displacement parameters (Å^2^)

**Table:**

	*x*	*y*	*z*	*U*_iso_*/*U*_eq_	
Co1	0.81347 (4)	0.21324 (2)	0.37752 (3)	0.01963 (9)	
Cl1	1.05621 (8)	0.10160 (5)	0.37941 (7)	0.03569 (16)	
Cl2	1.44553 (8)	0.40796 (6)	0.37507 (7)	0.03951 (17)	
N1	0.9146 (3)	0.34849 (16)	0.3746 (2)	0.0300 (5)	
C1	1.0619 (3)	0.3624 (2)	0.3717 (3)	0.0328 (6)	
H1	1.1328	0.3109	0.3701	0.039*	
N2	1.0955 (3)	0.45971 (18)	0.3714 (2)	0.0401 (6)	
H2N	1.1848	0.4849	0.3702	0.048*	
C2	0.9645 (3)	0.5127 (2)	0.3734 (3)	0.0435 (7)	
H2	0.9539	0.5822	0.3737	0.052*	
C3	0.8525 (3)	0.4432 (2)	0.3748 (3)	0.0385 (7)	
H3	0.7498	0.4575	0.3757	0.046*	
N3	0.8045 (4)	0.22115 (18)	0.52089 (16)	0.0319 (6)	
C4	0.8591 (4)	0.1555 (3)	0.5825 (2)	0.0344 (9)	
H4	0.9140	0.0985	0.5658	0.041*	
N4	0.8246 (4)	0.1822 (2)	0.67242 (18)	0.0415 (7)	
H4N	0.8488	0.1499	0.7237	0.050*	
C5	0.7453 (5)	0.2690 (3)	0.6690 (2)	0.0443 (10)	
H5	0.7074	0.3049	0.7210	0.053*	
C6	0.7323 (5)	0.2928 (3)	0.5757 (2)	0.0385 (9)	
H6	0.6823	0.3489	0.5518	0.046*	
N5	0.8038 (3)	0.21234 (18)	0.23384 (16)	0.0289 (5)	
C7	0.8709 (4)	0.1515 (3)	0.1730 (2)	0.0357 (9)	
H7	0.9358	0.0996	0.1903	0.043*	
N6	0.8326 (4)	0.1746 (2)	0.08285 (18)	0.0391 (7)	
H6N	0.8641	0.1450	0.0317	0.047*	
C8	0.7344 (5)	0.2537 (3)	0.0865 (2)	0.0417 (9)	
H8	0.6885	0.2855	0.0346	0.050*	
C9	0.7177 (5)	0.2762 (2)	0.1793 (2)	0.0363 (9)	
H9	0.6569	0.3273	0.2033	0.044*	
N7	0.6433 (3)	0.11427 (17)	0.3800 (2)	0.0308 (5)	
C10	0.4975 (3)	0.1353 (2)	0.3745 (3)	0.0391 (7)	
H10	0.4577	0.1999	0.3716	0.047*	
N8	0.4150 (3)	0.05047 (19)	0.3737 (3)	0.0429 (6)	
H8N	0.3184	0.0467	0.3702	0.051*	
C11	0.5122 (3)	−0.0281 (2)	0.3794 (3)	0.0444 (8)	
H11	0.4874	−0.0960	0.3805	0.053*	
C12	0.6517 (3)	0.0117 (2)	0.3829 (3)	0.0396 (7)	
H12	0.7406	−0.0252	0.3868	0.048*	

### Atomic displacement parameters (Å^2^)

**Table:**

	*U*^11^	*U*^22^	*U*^33^	*U*^12^	*U*^13^	*U*^23^
Co1	0.02202 (16)	0.02129 (15)	0.01558 (15)	−0.00434 (13)	0.00026 (19)	0.00021 (16)
Cl1	0.0317 (3)	0.0368 (4)	0.0385 (4)	0.0067 (3)	−0.0006 (5)	0.0032 (4)
Cl2	0.0337 (4)	0.0519 (4)	0.0329 (4)	−0.0094 (3)	−0.0003 (4)	0.0026 (4)
N1	0.0318 (11)	0.0291 (11)	0.0291 (11)	−0.0025 (9)	−0.0009 (15)	0.0017 (13)
C1	0.0323 (14)	0.0301 (14)	0.0358 (15)	0.0003 (12)	0.003 (2)	0.0029 (15)
N2	0.0310 (12)	0.0395 (14)	0.0497 (15)	−0.0106 (10)	0.0006 (17)	−0.0032 (16)
C2	0.0406 (16)	0.0286 (14)	0.061 (2)	−0.0019 (12)	−0.001 (2)	−0.0037 (19)
C3	0.0309 (15)	0.0322 (14)	0.0524 (17)	0.0003 (11)	0.001 (2)	0.0010 (19)
N3	0.0340 (15)	0.0350 (14)	0.0268 (12)	−0.0055 (12)	−0.0009 (13)	0.0024 (10)
C4	0.034 (2)	0.0409 (19)	0.0287 (15)	−0.0006 (16)	−0.0017 (14)	0.0023 (14)
N4	0.0365 (18)	0.0619 (19)	0.0260 (13)	0.0002 (18)	−0.0022 (15)	0.0070 (13)
C5	0.047 (2)	0.056 (2)	0.0303 (17)	0.0025 (19)	0.0028 (17)	−0.0074 (15)
C6	0.038 (2)	0.0402 (19)	0.0370 (17)	0.0080 (16)	0.0019 (15)	−0.0027 (14)
N5	0.0284 (14)	0.0297 (13)	0.0288 (12)	−0.0031 (11)	0.0009 (12)	0.0008 (10)
C7	0.037 (2)	0.0356 (18)	0.0345 (17)	−0.0011 (16)	−0.0030 (15)	−0.0038 (14)
N6	0.0409 (18)	0.0505 (17)	0.0259 (13)	−0.0045 (16)	0.0028 (13)	−0.0086 (12)
C8	0.052 (3)	0.0434 (18)	0.0302 (15)	−0.0002 (17)	−0.0072 (16)	0.0062 (14)
C9	0.040 (2)	0.0346 (18)	0.0339 (16)	0.0012 (15)	−0.0026 (16)	0.0025 (12)
N7	0.0311 (12)	0.0318 (12)	0.0294 (12)	−0.0015 (9)	0.0022 (15)	−0.0004 (12)
C10	0.0381 (16)	0.0370 (16)	0.0422 (17)	0.0018 (12)	−0.001 (2)	0.0020 (18)
N8	0.0277 (12)	0.0532 (15)	0.0477 (15)	−0.0080 (11)	0.0006 (19)	−0.0023 (18)
C11	0.0469 (17)	0.0321 (16)	0.054 (2)	−0.0125 (13)	0.004 (2)	−0.0006 (19)
C12	0.0359 (15)	0.0316 (14)	0.0513 (18)	0.0029 (12)	−0.001 (2)	−0.0004 (16)

### Geometric parameters (Å, º)

**Table:**

Co1—N3	1.999 (2)	C5—H5	0.9300
Co1—N5	2.001 (2)	C6—H6	0.9300
Co1—N7	2.003 (2)	N5—C7	1.314 (4)
Co1—N1	2.011 (2)	N5—C9	1.372 (4)
Co1—Cl1	2.6151 (7)	C7—N6	1.336 (4)
N1—C1	1.319 (4)	C7—H7	0.9300
N1—C3	1.375 (3)	N6—C8	1.367 (5)
C1—N2	1.328 (4)	N6—H6N	0.8600
C1—H1	0.9300	C8—C9	1.334 (4)
N2—C2	1.358 (4)	C8—H8	0.9300
N2—H2N	0.8600	C9—H9	0.9300
C2—C3	1.357 (4)	N7—C10	1.324 (4)
C2—H2	0.9300	N7—C12	1.368 (4)
C3—H3	0.9300	C10—N8	1.345 (4)
N3—C4	1.316 (4)	C10—H10	0.9300
N3—C6	1.379 (4)	N8—C11	1.357 (4)
C4—N4	1.336 (4)	N8—H8N	0.8600
C4—H4	0.9300	C11—C12	1.346 (4)
N4—C5	1.352 (5)	C11—H11	0.9300
N4—H4N	0.8600	C12—H12	0.9300
C5—C6	1.341 (5)		
			
N3—Co1—N5	174.57 (9)	C6—C5—H5	126.9
N3—Co1—N7	89.27 (12)	N4—C5—H5	126.9
N5—Co1—N7	88.92 (11)	C5—C6—N3	109.4 (3)
N3—Co1—N1	89.46 (11)	C5—C6—H6	125.3
N5—Co1—N1	90.26 (11)	N3—C6—H6	125.3
N7—Co1—N1	157.60 (9)	C7—N5—C9	106.1 (3)
N3—Co1—Cl1	93.01 (9)	C7—N5—Co1	128.9 (2)
N5—Co1—Cl1	92.40 (8)	C9—N5—Co1	125.0 (2)
N7—Co1—Cl1	104.28 (7)	N5—C7—N6	110.4 (3)
N1—Co1—Cl1	98.13 (7)	N5—C7—H7	124.8
C1—N1—C3	105.5 (2)	N6—C7—H7	124.8
C1—N1—Co1	124.59 (18)	C7—N6—C8	107.8 (3)
C3—N1—Co1	129.90 (19)	C7—N6—H6N	126.1
N1—C1—N2	111.0 (3)	C8—N6—H6N	126.1
N1—C1—H1	124.5	C9—C8—N6	106.2 (3)
N2—C1—H1	124.5	C9—C8—H8	126.9
C1—N2—C2	108.3 (2)	N6—C8—H8	126.9
C1—N2—H2N	125.8	C8—C9—N5	109.6 (3)
C2—N2—H2N	125.8	C8—C9—H9	125.2
C3—C2—N2	105.8 (2)	N5—C9—H9	125.2
C3—C2—H2	127.1	C10—N7—C12	105.4 (3)
N2—C2—H2	127.1	C10—N7—Co1	126.5 (2)
C2—C3—N1	109.3 (3)	C12—N7—Co1	128.0 (2)
C2—C3—H3	125.3	N7—C10—N8	110.7 (3)
N1—C3—H3	125.3	N7—C10—H10	124.6
C4—N3—C6	105.6 (3)	N8—C10—H10	124.6
C4—N3—Co1	126.9 (2)	C10—N8—C11	107.5 (2)
C6—N3—Co1	127.4 (2)	C10—N8—H8N	126.2
N3—C4—N4	110.4 (3)	C11—N8—H8N	126.2
N3—C4—H4	124.8	C12—C11—N8	106.4 (3)
N4—C4—H4	124.8	C12—C11—H11	126.8
C4—N4—C5	108.3 (3)	N8—C11—H11	126.8
C4—N4—H4N	125.9	C11—C12—N7	109.9 (3)
C5—N4—H4N	125.9	C11—C12—H12	125.0
C6—C5—N4	106.3 (3)	N7—C12—H12	125.0
			
C3—N1—C1—N2	0.9 (5)	C9—N5—C7—N6	0.7 (4)
Co1—N1—C1—N2	−179.3 (2)	Co1—N5—C7—N6	178.6 (2)
N1—C1—N2—C2	−0.5 (5)	N5—C7—N6—C8	−0.7 (5)
C1—N2—C2—C3	−0.1 (5)	C7—N6—C8—C9	0.4 (5)
N2—C2—C3—N1	0.6 (5)	N6—C8—C9—N5	0.0 (5)
C1—N1—C3—C2	−0.9 (5)	C7—N5—C9—C8	−0.4 (5)
Co1—N1—C3—C2	179.3 (3)	Co1—N5—C9—C8	−178.4 (3)
C6—N3—C4—N4	0.1 (4)	C12—N7—C10—N8	−0.1 (5)
Co1—N3—C4—N4	−176.3 (3)	Co1—N7—C10—N8	177.0 (2)
N3—C4—N4—C5	−0.4 (5)	N7—C10—N8—C11	0.3 (5)
C4—N4—C5—C6	0.5 (5)	C10—N8—C11—C12	−0.4 (5)
N4—C5—C6—N3	−0.4 (5)	N8—C11—C12—N7	0.3 (5)
C4—N3—C6—C5	0.2 (5)	C10—N7—C12—C11	−0.1 (5)
Co1—N3—C6—C5	176.6 (3)	Co1—N7—C12—C11	−177.2 (3)

### Hydrogen-bond geometry (Å, º)

Im3 = centroïde du cycle imidazole N5/N6/C7-C9

**Table:**

*D*—H···*A*	*D*—H	H···*A*	*D*···*A*	*D*—H···*A*
N2—H2*N*···Cl2	0.86	2.53	3.180 (3)	133
N4—H4*N*···Cl2^i^	0.86	2.40	3.247 (3)	168
N6—H6*N*···Cl2^ii^	0.86	2.40	3.251 (3)	169
N8—H8*N*···Cl1^iii^	0.86	2.44	3.254 (3)	158
C3—H3···Cl2^iii^	0.93	2.78	3.639 (3)	154
C4—H4···Cl1^iv^	0.93	2.78	3.541 (4)	140
C10—H10···Cl2^iii^	0.93	2.77	3.657 (3)	160
C2—H2···Im3^v^	0.93	2.82	3.526 (3)	134

Symmetry codes: (i) *x*−1/2, −*y*+1/2, *z*+1/2; (ii) *x*−1/2, −*y*+1/2, *z*−1/2; (iii) *x*−1, *y*, *z*; (iv) −*x*+2, −*y*, −*z*+1; (v) −*x*+3/2, *y*+1/2, −*z*+1/2.
